# Sweet-liking and sugar supplementation as innovative components in substance use disorder treatment: A systematic review

**DOI:** 10.1177/02698811251319454

**Published:** 2025-02-13

**Authors:** Jan van Amsterdam, Wim van den Brink

**Affiliations:** 1 Department of Psychiatry, Amsterdam UMC, University of Amsterdam, Amsterdam, The Netherlands; 2 Amsterdam Neuroscience, Research Program Compulsivity, Impulsivity and Attention, Amsterdam, The Netherlands

**Keywords:** Alcohol dependence, nicotine dependence, sugar, sweet, sweet-liking, abstinence, relapse

## Abstract

**Objective:**

Substance use disorders are a major global public health concern. While a wide range of psychotherapies and pharmacotherapies are available for their treatment, efficacy is limited and many patients fail to benefit from these treatments. Like addictive substances, sugar seems to trigger the dopaminergic reward centre, and sweet-liking might be a modifier of substance use disorder treatment.

**Method:**

Systematic review to summarize the role of sugar and sugar-liking in addiction and addiction treatment.

**Results:**

Evidence from both preclinical and clinical studies suggests that a certain portion of the population has a genetic predisposition for sweet-liking, which might be related to a higher risk for substance use and dependence. Regarding nicotine dependence, glucose supplementation prior to or during smoking cessation rapidly mitigates withdrawal symptoms and increases smoking abstinence rates during nicotine replacement therapy. In alcohol dependence, sweet-liking patients encounter more challenges in achieving abstinence than sweet-disliking patients. In addition, sweet-liking patients with high cravings demonstrate higher abstinence rates than sweet-disliking patients. Finally, sweet-liking is associated with successful outcomes of naltrexone treatment in patients with an alcohol use disorder.

**Conclusion:**

These findings present promising new challenges and opportunities to fine-tune and optimize treatment protocols in addiction care.

## Introduction

Substance use disorders (SUDs) are a significant public health concern worldwide. For example, in the United States, alcohol use disorders (AUDs) are the third leading preventable cause of death (Mokdad et al., 2004) and in 2021 there were more than 107,000 drug overdose deaths (CDC, 2020). While various psychotherapies and pharmacotherapies are available for the treatment of SUD, their efficacy is limited, prompting an ongoing search for alternative or additional treatments. Personalized addiction treatment considering the sweet-liking (SL) phenotype and/or the use of sugar supplementation may constitute such treatment components.

### SUD and SL

As early as 1970, it was observed that smokers added more sugar to their hot drinks (coffee and tea) than non-smokers or former smokers (Bennett et al., 1970), and individuals dependent on alcohol or drugs had a higher preference for sweets than their non-dependent peers (Fortuna, 2010; Kampov-Polevoy et al., 2003, 2004). The fellowship ‘Alcoholics Anonymous’ traditionally advises new members to carry candies with them to help suppress the urge to drink (Alcoholics Anonymous, 2020), suggesting that sugars reduce withdrawal symptoms and/or substitute the rewarding effects of addictive substances when their use is acutely discontinued.

Both the ‘SL’ phenotype, that is, those who like sucrose concentrations from 0.83 up to 2.0 M (Kampov-Polevoy et al., 1997, 1998), and ‘high’ sugar consumption have been linked to the use of addictive substances and the development of SUD. For instance, the propensity of children aged 5–9 years to consume sugar and fat predicted alcohol use in early adolescence (Mehlig et al., 2018).

These claims are supported by findings from preclinical studies showing that sugar and sweetness can induce reward and craving comparable in magnitude to those induced by addictive drugs (Ahmed et al., 2013). For instance, rodent studies have revealed that very sweet tastes, such as saccharin, can substitute for addictive drugs like cocaine and may even be more rewarding and attractive for these animals (Ahmed et al., 2013; Lenoir et al., 2007). Interestingly, the preference for saccharin mainly emerged in rats that had originally developed a strong preference for the cocaine-rewarded lever (Lenoir et al., 2007). Under certain circumstances, sugar-liking may even evolve into excessive consumption, which may be harmful thus meeting at least one important SUD criterion: continued (excessive) use despite negative consequences. Accumulating data from animal experiments, particularly in rodents, have shown strong parallels between sugar overconsumption and drug addiction (Lenoir et al., 2007) and provide ample evidence for the existence of sugar addiction (Avena et al., 2008).

### Shared genetic predisposition for substance use and sugar-liking

Both SUD and sugar-liking are strongly influenced by genetic factors (Agrawal and Lynskey, 2008; Jacob et al., 2003). For instance, alcohol dependence has a substantial genetic component (Gelernter, 1995), as evidenced by family, twin, half-sibling and adoption studies of individuals with AUD (Ferguson and Goldberg, 1997). For SUD, including nicotine dependence (Vink et al., 2005) and alcohol dependence (Ferguson and Goldberg, 1997; Verhulst et al., 2015), the contribution of hereditary factors is at least 50% (Kendler et al., 2000). Similar rates of heritability (49%–53%) have been reported for SL and the frequency of consumption of sweet foods (Keskitalo et al., 2007; Pallister et al., 2015). A genetic study among 8586 twins, investigating whether sugar consumption and substance use share genetic or environmental risk factors, showed that the (moderate) phenotypic association between high sugar consumption and high substance use (*r* = 0.2) was explained for 59% by shared genetic factors and for 41% by unique environmental factors, such as (early) family life, upbringing and social situations (Treur et al., 2016), suggesting common underlying neuronal pathways of drug addiction and sugar-liking. In addition, the hedonic value of SL is associated with a genetic risk for AUD (Kampov-Polevoy et al., 1997, 2004; Pepino and Mennella, 2007; Wronski et al., 2007). For example, a family history of AUD predicted SL with an odds ratio of 2.5 (Kampov-Polevoy et al., 2001, 2003), though others failed to demonstrate such an association (Kranzler et al., 2001; Scinska et al., 2001). Finally, a Korean study found an association of the *TAS1R3* rs307355 CT gene variation, encoding for sweet taste receptors, with SL (Bachmanov et al., 2011) and heavy drinking (OR: 1.53, 95% CI: 1.06–2.19) (Choi et al., 2017). In summary, a portion of the population has a genetic predisposition for SL which seems to be associated with a higher risk for SUD.

### Shared pathways for substance use and sugar-liking

The results of pre-clinical studies performed in rats selectively bred for high or low sweet preference (saccharin intake; Carroll et al., 2008) support the putative link between sweet consumption and drug self-administration possibly mediated by genetic differences in emotionality, impulsivity and novelty reactivity. Furthermore, Peciña and Berridge identified a small but specific ‘hot spot’ in the nucleus accumbens of rats where an enkephalin-analogue could elicit µ-opioid ‘sucrose liking’ (Peciña and Berridge, 2000, 2005). However, the results of another rodent study showed an apparent genetic dissection of alcohol and sweet liking, considering that a minority of outbred rats, characterized by a certain constellation of behavioural traits, preferred to continue self-administration of alcohol over an available high-value alternative (e.g. sugar) suggesting that subpopulations are at risk to transit from controlled to compulsive alcohol use (Augier et al., 2018).

Reward sensitivity is positively associated with alcohol and cigarette consumption (Tapper et al., 2015) and individuals with high reward sensitivity also show a preference for sweet and fatty foods, high alcohol consumption, binge eating and other addictive (like) behaviours (Davis, 2013; Tapper et al., 2015). Considerable research has been dedicated to assessing the impact of sugar on the neural pathways that mediate reward. It has been proposed that the relationship between SL and alcohol consumption is mediated by common neurochemical reward mechanisms, including opioid, dopaminergic and serotonergic influences (Kampov-Polevoy et al., 1999). Indeed, pre-clinical studies have provided ample evidence for the similarity between sugar overconsumption and drug addiction (Volkow and Wise, 2005). It is well established that drugs of abuse (alcohol, nicotine, cocaine, (meth)amphetamine, etc.) stimulate dopamine signalling in the ventral striatum/nucleus accumbens, a brain signalling pathway critically involved in reward processing and SUD.

Likewise, rodent studies have demonstrated that ingestion of foods or fluids enriched in sugars can trigger the release of dopamine within the ventral striatum (Avena et al., 2006; Hajnal et al., 2004; Rada et al., 2005). Furthermore, both cross-tolerance (Lieblich et al., 1983) and cross-dependence (Colantuoni et al., 2002) have been observed between sugars and drugs of abuse.

Furthermore, functional magnetic resonance imaging in healthy volunteers (*n* = 74) showed that a sucrose challenge produced robust activation in the primary gustatory cortex, ventral insula, amygdala and ventral striatum. Interestingly, a low (0.10 M) concentration of sucrose elicited greater bilateral amygdala activation in subjects with a family history of alcoholism compared to their peers without a family history of alcoholism (Eiler et al., 2018). Finally, a systematic review provided tentative evidence for activation of the reward-related caudate nucleus by the sweet taste of caloric sugars (Roberts et al., 2020). In this respect, it is of interest to note that the opioid antagonist naltrexone decreased the pleasantness (‘liking’) of sucrose solutions in healthy subjects (Eikemo et al., 2016).

In summary, there is overlap in the dopaminergic reward circuitry associated with ‘SL’ and the effect of addictive substances, considering that sugar consumption seems to trigger the reward circuit.

In this systematic review, we investigate (a) whether SL individuals and sweet-disliking (SDL) individuals respond differently to abstinence-oriented treatments in people with SUD and (b) whether sugar consumption, as an appreciated substitute, may mitigate the mental problems of dependence following acute discontinuation of drug use.

## Methods

**Figure 1.**
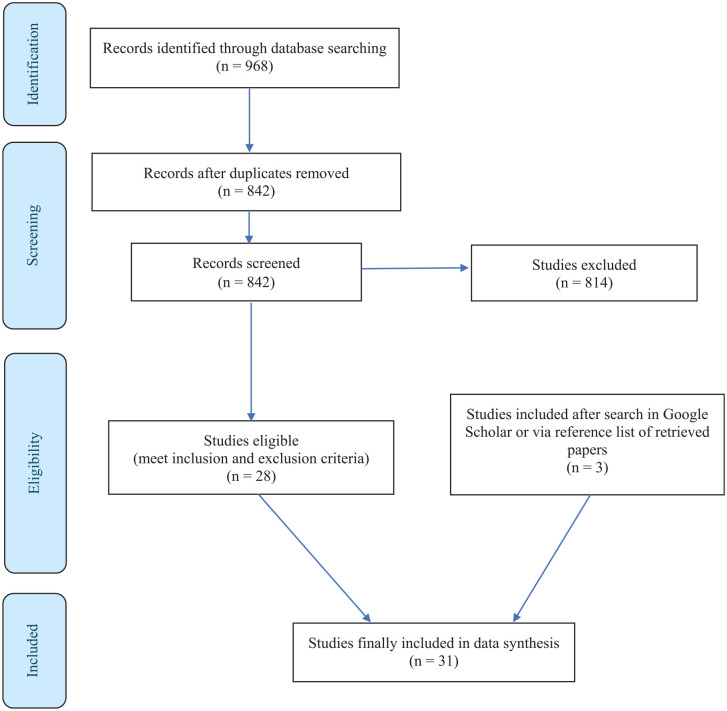
PRISMA flow diagram.

We aimed to provide an up-to-date and in-depth examination of the literature on the impacts of sugar on withdrawal and abstinence from addictive substances, particularly nicotine and alcohol. Using the Preferred Reporting Items for Systematic reviews and Meta-Analyses (PRISMA) protocol, a systematic review was therefore performed in PubMed and Embase on 15 August 2024 to retrieve relevant studies, including those published or accepted but ahead of print. Inclusion criteria were as follows: studies to SUD in relation to either SL phenotype or sugar supplementation as intervention were required to be conducted in nicotine-dependent subjects, in subjects with an AUD assessed according to DSM-III-R, DSM-IV, DSM-V or ICD-10 criteria of the third-revised, fourth and fifth edition of the Diagnostic and Statistical Manual of Mental Disorders (DSM-III-R, DSM-IV, DSM-V) or the tenth edition of the International Classification of Diseases (ICD-10) criteria or in subjects with heavy drinking behaviour as assessed by the *Alcohol* Use Disorder Identification Test (AUDIT) score. Excluded were studies on obesity, cancer and diabetes, as well as case reports, editorials and commentaries. The selection of eligible studies was independently performed by JvA and WvdB in two rounds. A total of 968 studies were identified from the initial search and 842 articles remained after duplicates were removed. These 842 studies were further processed, that is, the title and abstract were screened to determine eligibility using the above inclusion criteria which resulted in 28 papers. In a second round, three additional studies were included after a search in Google Scholar or via the reference list of retrieved papers. Tables 1 and 2 depict the 31 eligible studies retrieved. See Figure 1 for the PRISMA flow diagram and the supplement for the search string and PRISMA checklist.

**Table 1.** Results of studies on the association between phenotypes, like sweet-liking (SL), novelty seeking (NS), sensitivity to the impairing effects of alcohol and alcohol and cocaine-related problems, and abstinence rate.

Subjects	Comparators	Main outcome	References
Descriptive studies			
20 AUD patients who met DSM-IIIR criteria, abstinent for ⩾28 days and 37 CTR	Sweet-liking (SL) by AUD patients and controls	65% of AUD patients were SL compared with only 16% of controls	Kampov-Polevoy et al. (1997)
26 AUD patients who met DSM-IIIR criteria and 52 CTR	SL, novelty seeking (NS) and propensity to avoid harm as predictors of AUD	SL, harm avoidance and NS predicted AUD versus non-AUD status at 65% sensitivity and 94% specificity, with a correct classification in 85% of subjects	Kampov-Polevoy et al. (1998)
32 AUD patients who met DSM-IIIR criteria and 25 CTR	SL by AUD patients and controls	On the fifth day after admission, 46% of AUD patients were SL compared with only 12% of controls (*p* = 0.048)	Kampov-Polevoy et al. (2001)
150 young adults	NS and SL phenotypes vs AUDIT score that is, alcohol consumption and alcohol-related problems	High NS, but not SL, was positively associated with alcohol consumption and alcohol problems. Strong synergistic interaction for NS in SL phenotype on alcohol problems (OR = 20.15; *p* = 0.0001) compared to those with low NS and SDL	Kampov-Polevoy et al. (2014)
145 young adults	Subtypes of NS, SL and initial insensitivity to the impairment by alcohol vs AUDIT score	NS, SL and initial insensitivity to the impairing effects of alcohol independently and synergistically increased the AUDIT score, explaining together 35.8% of AUDIT variance	Kampov-Polevoy et al. (2022)
158 healthy subjects who did not meet DSM-III-R criteria	NS and SL phenotypes vs alcohol-related problems	NS score, but not SL/SDL status, was positively correlated with drinks per month (*p* = 0.005) and drinks per drinking day (*p* = 0.02). Both high NS and SL phenotyping were associated with alcohol-related problems (NS: OR = 5.3, *p* = 0.0016; SL/SDL: OR = 5.8, *p* = 0.0001). Combined SL status and high NS were highly associated with having alcohol-related problems (OR: 27.5; *p* < 0.0001)	Lange et al. (2010)
55 heavy drinkers^a^ (*n* = 52 met DSM-IV criteria)	SW and alcohol cravings^b^	More craving in SL (*n* = 20) compared to SDL (*n* = 35; *t*(42) = 3.82; *p* = 0.0004). No differences between SL and SDL in the number of heavy drinking days	Bouhlal et al. (2018)
57 healthy males and 136 healthy females	SL/SDL status and alcohol consumption	In males, but not in females, SL showed a 24% higher alcohol consumption per month (*F*(1) = 3.94, *p* = 0.05) than SDL. Alcohol consumption (*t*(191) = 1.97, *p* = 0.23) or SL classification (*t*(191) = 1.97, *p* = 0.41) did not vary with the AUDIT cut-off score of ⩾8 (i.e., high vs low risk of hazardous drinking)	Robb and Pickering (2019)
30 male AUD patients (13 with a positive family history (PFH) of alcohol dependence)	Detoxification and desire for sweets	At 6 weeks after detoxification from alcohol, patients with PFH of alcohol dependence did not relapse earlier but they had a stronger desire to eat sweets	Junghanns et al. (2005)
122 non-alcoholic subjects; 58 had a paternal history of alcoholism according to DSM-III-R criteria	SL as a marker of alcoholism risk	Irrespective of gender, both subjects with and those without a paternal history of alcoholism preferred a 0.42 M sucrose solution	Kranzler et al. (2001)
AUD patients (*n* = 68) and CTR (*n* = 36)	SL and alcohol abstinence	At baseline, more of the AUD subjects (71%) expressed maximal SL compared to 37% of controls (*t*(63) = 2.86, *p* = 0.0006). However, by 6 months, this difference had disappeared (*t*(55) = 0.69, *p* = 0.4922)	Krahn et al. (2006)
30 abstinent male AUD patients	SL and alcohol craving	No difference in craving for sweets between AUD patients (mean ± SEM: 22.7 ± 4.5) and controls (15.4 ± 4.3; *t* = 1.18, *p* > 0.05)	Bogucka-Bonikowska et al. (2001)
45 AUD patients who met ICD-10 criteria and 33 CTR	SL by AUD patients and controls	No difference between male AUD patients and controls with respect to SL	Wronski et al. (2007)
100 healthy young adults	SL and alcohol consumption	No correlation between SL ratings and any measures of alcohol consumption, including AUDIT measures (*p* > 0.10)	Weafer et al. (2014)
16 patients with cocaine dependence and 16 CTR	Preference for, and the degree of sweetness of sugar solutions	Subjects with cocaine use disorder showed more often a preference for the highest sucrose concentration (0.83 M) compared to controls	Janowsky et al. (2003)
Intervention studies			
Open-label study, 40 AUD patients, randomized as SL (*n* = 15) and SDL (*n* = 25)	NTX (50 mg daily) plus four sessions of MET for 12 weeks	Pre-treatment, SL took ∼40% longer to achieve 3 days of abstinence compared to SDL (*p* = 0.02). During treatment, SDL had more abstinent days compared to SL (48% vs 30%; *p* = 0.034). In subjects with high craving: SL demonstrated higher rates of abstinent days compared to SDL (*p* < 0.001)	Garbutt et al. (2009)
RCT in AUD patients, randomized as SL (*n* = 22) or SDL (*n* = 58) and by pre-treatment high (*n* = 40) or low (*n* = 40) craving for alcohol	NTX (50 mg/d daily) or PLC with 2–4 times per month brief counselling for 12 weeks	No effect of NTX on heavy drinking (4.8 fewer heavy drinking days; *p* = 0.07), but SL phenotype moderated this NTX effect (6.1 fewer heavy drinking days; *p* = 0.02) and abstinence (10.0 more abstinent days; *p* = 0.02). The SL subgroup with high craving demonstrated a marked response to NTX compared with placebo (17.1 fewer heavy drinking days; *p* < 0.001). Compared with PLC, the combination of the SL phenotype and high craving was associated with a strong response to NTX, with 17.1 fewer heavy drinking days (*p* <0.001) and 28.8 more abstinent days (*p* = 0.004)	Garbutt et al. (2016)
RCT in 78 AUD patients	Treatment for 32 weeks with NTX (50 mg daily) or PLC	In NTX, but not in the PLC group, SL was significantly related to successful treatment outcome	Laaksonen et al. (2011)
Healthy subjects (36 men; 34 women)	At random d-amphetamine (20 mg) or placebo	In women, but not in men, SL reported significantly greater amphetamine-induced euphoria than did SDL	Weafer et al. (2017)

CTR: controls; SL: sweet-likers; SDL: sweet-dislikers; AUD: alcohol use disorder assessed according to DSM-IV criteria or as stated otherwise; NS: novelty seeking; OR: odd ratio; AUDIT: *alcohol* use disorder identification test; MET: motivational enhancement therapy; NTX: naltrexone; PLC: placebo.

a Heavy drinking was defined as >14 drinks per week for men and >7 drinks per week for women.

b Craving was assessed using the Obsessive-Compulsive Drinking Scale (OCDS).

**Table 2.** Results of intervention studies in clinical samples and healthy subjects to the effect of treatment with sugars on abstinence from different substances.

Subjects	Treatment	Main outcome	References
Nicotine dependence			
RCT, 20 smokers abstinent for 1 week	Dextrose up to 20 g per day or sorbitol (PLC) ad lib. in the next week	Significant reduction in ratings of urges to smoke and craving after one additional week of abstinence	West et al. (1990)
RCT, 308 smokers	For 4 weeks: dextrose ⩽ 45 g daily, NRT (nicotine patch, 15 mg) or PLC	Abstinence rate after 4 weeks: dextrose + NRT (49%); PLC + NRT (36%); dextrose + PLC patch (44%) and PLC tablet + placebo patch (30%). The 13% difference was significant (*p* < 0.01, one-tailed)	West and Willis (1998)
RCT, 38 smokers abstained from smoking from the previous evening	One dose of 12 g glucose or PLC ad lib. on the next morning	Less craving during 20 min after consumption of glucose (scored every 5 min)	West et al. (1999)
RCT, 928 smokers	Glucose or sorbitol (PLC) ad lib. plus psychological support; 474 smokers also received also NRT, bupropion or both	No effect on 6-month continuous abstinence (14.6% vs 13.4% for glucose and PLC, resp.). However, a significant interaction with a glucose effect was observed in smokers also receiving other medication (18.2% vs 12.6%, *p* < 0.05) suggesting a possible effect of glucose as an adjunct to NRT or bupropion	West et al. (2010)
Cross-over in 67 female smokers	50 g sucrose or 0.33 g aspartame	Sucrose decreased abstinence-induced drowsiness, anxiety and preference for high-calorie food, but not restlessness, irritability and concentration difficulties, 12 h after abstinence	Helmers and Young (1998)
RCT in smokers (31 on bupropion; 44 on NRT) abstinent for 1 week	Glucose (12 g) or PLC	Withdrawal was rated at 5-min intervals during 20 min after glucose/PLC. In bupropion users, glucose reduced irritability (34% reduction; *p* = 0.03), but not restlessness and total withdrawal discomfort. No differences in the NRT group	McRobbie and Hajek (2004)
RCT, 40 smokers	Glucose (12 g) or PLC	No effect on craving and withdrawal symptoms at 5-min intervals for 20 min after 12 h of tobacco abstinence	Harakas and Foulds (2002)
Double-blind crossover study in tobacco smokers (*n* = 12) abstinent for 12 h	Aspartame (PLC) or 32.5 g glucose or 75 g glucose	Tobacco craving increased with aspartame but not with glucose (condition by time interaction: Wilks’ λ = 0.144, *F*(6, 6) = 5.95, *p* = 0.024). No significant differences at 1–3 h, but tobacco craving at 5 h was significantly higher after aspartame than after 32.5 g (*p* = 0.04) or 75 g (*p* = 0.012) glucose. Only minor differences were seen in the withdrawal	Berlin et al. (2005)
Alcohol dependence			
RCT in AUD patients (*n* = 68) and controls (*n* = 36)	Three different dietary advice (eat sweets; eat a balanced diet; avoid sweets)	At the 6-month follow-up, no effect of a specific dietary recommendation on urges to drink or alcohol consumption. Importantly, AUD patients who failed to maintain abstinence through 6 months were much more likely to be SL phenotype than were those who maintained abstinence	Krahn et al. (2006)
Single blinded study in AUD patients (*n* = 150; AUDIT score: 8–15)	Subject were assigned to eat sweets (*n* = 60), eat calorie-equivalent bland food (*n* = 60) or watch a video (*n* = 30)	No between-group food effects on the rate of alcohol cravings, assessed via VAS at 15 min after exposure to a second alcohol cue	Cummings et al. (2020)
EMA-study in 25 AUD patients	Daily questionnaire (4 times/day) during 3 weeks following treatment	Consumption of sweets earlier in the day predicted higher alcohol cravings later in the day (*p* = 0.004)	Abrantes et al. (2022)
Prospective observational study in 49 AUD patients	Self-reported chocolate consumption at baseline (T1) and at 6 months (T4)	Still abstinent patients at T4 consumed 3-times more chocolate than non-abstainers, but this pattern was not associated with reduced craving	Stickel et al. (2016)
Newly sober AUD outpatients (*n* = 64)	Dietary intake versus length of sobriety	Those who consumed more overall carbohydrates and added twice as much table sugar to beverages stayed sober longer (*p* < 0.05). More specifically: those who remained sober for more than 30 days used on average almost 3 times more sugar per cup of beverage than those who remained sober for less than 30 days (*p* < 0.01)	Yung et al. (1983)

PLC: placebo; NRT: nicotine replacement therapy; AUD: alcohol use disorder according to DSM-IV criteria or as stated otherwise; EMA: ecological momentary assessment.

## Results

### SL and response to abstinence treatment

Most clinical studies make use of the sucrose preference test, a standardized SL test using various concentrations of aqueous sucrose solutions (0.05, 0.10, 0.21, 0.42, 0.83, 1.0 and 2.0 M). This test is often used to identify and distinguish individuals with an SL from an SDL phenotype. SL individuals note increasing pleasantness of sucrose and are defined as those who prefer the highest sucrose concentration typically being at 0.83 or 1.0 M, whereas SDL individuals do not like sucrose concentrations higher than 0.4 M (Kampov-Polevoy et al., 1997, 1998). The SDL phenotype is characterized by an increased preference for sucrose concentrations up to 0.4 M, followed by a progressive decline in preference. For comparison: Coca-Cola ClassicTM is a 0.33 M sugar solution and can, thus, be liked by both SL and SDL individuals. Though different phenotyping methods have been used, we did not identify a method distinctly superior to the one presented here (Iatridi et al., 2019).

#### Observational studies with the SD/SDL phenotype

Table 1 summarizes the results of descriptive studies on the association between phenotypes (SL vs SDL, novelty seeking, sensitivity to the impairing effects of alcohol) and alcohol-related problems with AUD patients meeting either ICD-10, DSM-III-R or DSM-IV criteria. In AUD patients abstinent for at least 28 days, AUD was associated with SL with 65% of AUD patients being SL compared to only 16% of SL in the control group, suggesting a positive association between the preference for strong sweets and AUD (Kampov-Polevoy et al., 1997). In a subsequent study, 26 male AUD patients and 52 male controls were tested for sweet preference, novelty seeking and the propensity to avoid harm. SL, harm avoidance and novelty-seeking predicted AUD versus non-AUD status with 65% sensitivity and 94% specificity, with a correct classification in 85% of subjects (Kampov-Polevoy et al., 1998). Two other studies by this group performed in young adults (Kampov-Polevoy et al., 2014, 2022) included high novelty seeking (NS) and initial insensitivity to the impairing effects of alcohol (SRE-A) as covariates of the relation of SL/SDL phenotypes with alcohol consumption and alcohol-related problems. A strong synergistic interaction between NS and SL phenotype was seen (OR = 20.15; *p* = 0.0001) compared to those with low NS and SDL phenotype (Kampov-Polevoy et al., 2014). SL was associated with a higher AUDIT score (Kampov-Polevoy et al., 2022) and SL, NS and SRE-A synergistically increased the AUDIT score (Kampov-Polevoy et al., 2022). Furthermore, in another sample of 158 healthy subjects NS (OR = 5.3, *p* = 0.0016), SL/SDL (OR = 5.8, *p* = 0.0001) and combined SL status and high NS (OR: 27.5; *p* < 0.0001) were associated with alcohol-related problems (Lange et al., 2010).

The sucrose preference test was also used in a sample of 55 heavy drinkers (*n* = 52 with current AUD) to characterize subpopulations (Bouhlal et al., 2018). Two different groupings were used: grouping A consisted of 20 SL individuals preferring the sweetest solution 0.83 M and 35 SDL individuals who did not; grouping B consisted of 32 SL individuals who gave the highest pleasantness ratings to either the 0.42 M or the 0.83 M sucrose solutions, and 23 SDL individuals who did not. Based on grouping A, SL individuals had higher alcohol cravings than SDL individuals (*p* = 0.0004), but with grouping B no difference in alcohol cravings between SL and SDL was seen (*p* = 0.21). According to both groupings, SL and SDL individuals did not differ in the number of heavy drinking days or the average number of drinks per drinking day.

Sweet-taste appreciation of discs impregnated with 9, 90, or 900 g/l sucrose was assessed to classify 57 healthy males and 136 healthy females either as SL or SDL (Robb and Pickering, 2019). In males, but not in females, the SL classification showed a significant main effect on alcohol consumption (*p* = 0.05), with a higher alcohol consumption per month by SL (*M* = 3.56 ± 1.06 standard drinks) than by SDL (*M* = 2.86 ± 1.22). However, neither alcohol consumption (*p* = 0.23) nor SL classification (*p* = 0.41) was associated with the AUDIT cut-off score of ⩾8 (i.e., high vs low risk of hazardous drinking; Robb and Pickering, 2019).

At 6 weeks after detoxification from alcohol, patients with a positive family history (PFH) of alcohol dependence did not relapse earlier than their non-PFH peers but had a stronger desire to eat sweets (Junghanns et al., 2005). In a sample of 122 non-alcoholic individuals of whom 58 had a paternal history of alcoholism, SL was evaluated as a marker of alcoholism risk (Kranzler et al., 2001). It was shown that both subjects with and those without a paternal history of alcoholism preferred a 0.42 M sucrose solution refuting the hypothesis that sweet preference is a determinant of alcoholism risk (Kranzler et al., 2001). Krahn et al. (2006) demonstrated that, at baseline, patients with an AUD diagnosis were more likely than controls (71% vs 37%) to prefer highly sweet tastants, but this difference disappeared by 6 months of abstinence (Krahn et al., 2006). Others also failed to find an association between SL and AUD. For instance, male AUD patients did not differ from controls with regard to SL (Bogucka-Bonikowska et al., 2001; Weafer et al., 2017; Wronski et al., 2007).

Subjects with cocaine use disorder (CUD) showed a preference for the highest sucrose concentration (0.83 M) more often compared to controls (Janowsky et al., 2003). No data about SL/SDL phenotype in relation to nicotine dependence have been published.

#### Intervention studies on the effect of the SD/SDL phenotype

In an open-label study among 40 AUD patients, 15 patients were identified with the SL phenotype and 25 patients with the SDL phenotype and all were treated for 12 weeks with naltrexone (NTX, 50 mg daily) plus four sessions of motivational enhancement therapy (Garbutt et al., 2009). SL and SDL subjects achieved similar reductions in percent heavy drinking days with treatment. However, during treatment, SDL subjects had 48% abstinent days compared to only 30% for SL subjects (*p* = 0.034). Also, a significant (*p* < 0.001) interaction effect was found between the SL/SDL phenotype and pre-treatment craving: SL subjects with high craving demonstrated higher rates of percent abstinent days whereas SDL subjects with high craving demonstrated lower rates of percent abstinent days (Garbutt et al., 2009). In a randomized controlled trial (RCT) in AUD patients (*n* = 80), patients were pre-stratified according to their SL/SDL status (SL: *n* = 22; SDL: *n* = 58) and their pre-treatment alcohol craving level (high *n* = 40, low *n* = 40) and were treated for 12 weeks with NTX (50 mg/d daily) combined with 2–4 brief counselling sessions per month (Garbutt et al., 2016). A non-significant effect of naltrexone on heavy drinking was noted, but the SL phenotype moderated the effect of naltrexone on heavy drinking (6.1 fewer heavy drinking days; Cohen *d* = 0.58; *p* = 0.02) and abstinence (10.0 more abstinent days; Cohen *d* = 0.57; *p* = 0.02), and high craving moderated heavy drinking (7.1 fewer heavy drinking days; Cohen *d* = 0.66; *p* = 0.008). The combination of the SL phenotype and high craving was associated with a strong response to naltrexone, with 17.1 fewer heavy drinking days (Cohen *d* = 1.07; *p* < 0.001) and 28.8 more abstinent days (Cohen *d* = 0.72; *p* = 0.004) compared with placebo (Garbutt et al., 2016). Finally, the results of an RCT, performed in 78 AUD patients who were treated with either NTX (50 mg daily) or placebo for 32 weeks without prior detoxification, showed that in the NTX group but not in the placebo group, higher sweet preference was significantly related to successful treatment outcome, suggesting that sweet preference might be used as a predictor for better treatment results in AUD patients (Laaksonen et al., 2011).

With respect to stimulant drugs, the association between SL and sensitivity to amphetamine reward (20 mg) was tested in 70 healthy adults (36 men and 34 women). In women, but not in men, SL individuals reported significantly greater amphetamine-induced euphoria than did SDL individuals (Weafer et al., 2017). No data about SL/SDL phenotype in relation to nicotine dependence have been published.

In summary, SL seems to be associated with alcohol dependence and alcohol-related problems and some results suggest that SL/SDL phenotype impacts the responsiveness to NTX in actively drinking AUD patients. Individuals with an SL phenotype seem to have more problems attaining abstinence compared to SDL individuals. Interestingly, among those AUD patients who experienced high cravings, subjects with SL phenotype demonstrated higher rates of abstinent days compared to their SDL peers. The SL/SDL phenotype approach has not been evaluated in nicotine-dependent subjects.

#### Effect of sugar supplementation in addiction treatment

Patients in early recovery with substance use disorders have reported the substitution of sweets for substances to improve mood and soothe cravings (Cowan and Devine, 2008). Table 2 summarizes the results obtained in clinical trials investigating the effects of treatment with sugars on abstinence from different substances in smokers and AUD patients. The AUD patients described and specified in Table 2 met DSM-IV criteria or had an AUDIT score between 8 and 15.

#### Nicotine dependence

In one of their first RCTs to test the effect of sugar supplementation on reducing craving for cigarettes, West et al. (1990) administered either 12 g dextrose or sorbitol (placebo) ad lib to 20 clients attending a smokers clinic who had already been abstinent for 1 week. Glucose treatment significantly reduced ratings of urges to smoke and craving after 1 week of abstinence, suggesting that sugar supplementation and cigarette craving in smokers ready to give up smoking are related (West et al., 1990). In another RCT, 308 smokers received dextrose tablets (ad lib, up to 45 g daily) and 15 mg nicotine transdermal patches or placebo tablets and nicotine transdermal patches for 4 weeks (West and Willis, 1998). Four weeks after dextrose plus active patch, the abstinence rate was 49%, whereas it was 36% for placebo plus an active patch (*p* < 0.01). Abstinence rates for dextrose plus placebo patch and placebo tablet plus placebo patch were 44% and 30%, respectively. These results suggest that dextrose supplementation may be a simple aid in smoking cessation. A subsequent study on the effect of glucose on short-term abstinence showed that the desire to smoke after not smoking since the previous evening (*n* = 38) was lower within 20 min after chewing four 3-g glucose tablets compared to those chewing four placebo tablets (West et al., 1999). This result confirms a previous study which showed that 20 smokers abstinent for 2 weeks less craving for cigarettes after glucose up to 20 g per day in the second week compared to those receiving sorbitol (West et al., 1990). In a randomized double-blind placebo-controlled trial of glucose to aid smoking cessation, smokers attempting to stop (*n* = 928) were randomized to receive glucose or sorbitol (placebo) in addition to group-based psychological support (West et al., 2010). About half of the participants (*n* = 474) also received nicotine replacement therapy (NRT), bupropion or both. Smokers were seen weekly for 5 weeks and used glucose tablets ad lib, with a recommended minimum of 12 per day. The outcome showed no significant effect of glucose on 6-month continuous CO-verified abstinence rates (14.6% vs 13.4% abstinence for glucose and placebo, respectively). However, there was a significant effect of glucose in smokers also receiving other medication (18.2% vs 12.6%, *p* < 0.05), suggesting a possible interaction effect of glucose as an adjunct to NRT or bupropion (West et al., 2010). In another study in female smokers (*n* = 91), sucrose decreased abstinence-induced drowsiness and anxiety and the preference for foods high in carbohydrate and fat content, but it had no effect on other withdrawal symptoms, including restlessness, irritability and concentration difficulties (Helmers and Young, 1998). In an RCT performed in smokers abstinent for 1 week but receiving bupropion (*n* = 31; 150 mg twice daily) or NRT (*n* = 44), glucose (12 g) reduced irritability in bupropion users for 20 min (but not in NRT users) during abstinence, but glucose had no effect on restlessness and total withdrawal discomfort (McRobbie and Hajek, 2004). However, another double-blind, placebo-controlled, randomized study among 40 smokers failed to show an effect of chewing 12 g glucose tablets on the desire to smoke and on withdrawal symptoms after 12 h of tobacco abstinence (Harakas and Foulds, 2002).

In a small double-blind crossover trial (Berlin et al., 2005), 12 healthy regular smokers (5 women; ⩾15 cigarettes per day) received after an overnight (⩾8 h) abstinence 0.6 g aspartame (as placebo) or 32.5 or 75 g glucose per 200 ml tap water at 1-week intervals. Tobacco craving increased with aspartame but not with glucose (*p* = 0.024). No statistically significant differences occurred at 1–3 h, but tobacco craving at 5 h was significantly higher after aspartame than after 32.5 g (*p* = 0.04) or 75 g (*p* = 0.012) glucose. Only minor differences were seen for withdrawal. The authors concluded that glucose attenuated tobacco craving in temporarily abstinent smokers (Berlin et al., 2005).

In summary, a single dose of glucose seems to have a rapid and detectable effect on the alleviation of cravings in abstaining heavy smokers (West et al., 1990, 1999). In addition, dextrose (ad lib, up to 45 g daily) given for 4 weeks increased post-treatment smoking abstinence induced by NRT compared to NRT alone.

#### Alcohol dependence

In the study of Krahn et al. (2006), alcohol-dependent subjects who were in early abstinence were instructed to follow three different dietary advices (eat sweets; eat a balanced diet; avoid sweets). At the 6-month follow-up, no effect of dietary recommendations on urges to drink or alcohol consumption was observed. Importantly, AUD subjects who failed to maintain abstinence through 6 months were much more likely to belong to the SL phenotype than were those who maintained abstinence. Yet, this study did not assess actual sugar consumption (Krahn et al., 2006). In a single-blind study, after an alcohol cue, subjects with at-risk drinking (*n* = 150) were asked to eat sweets (*n* = 60), eat calorie-equivalent bland food (*n* = 60) or watch a video (*n* = 30), and were then exposed to a second alcohol cue. No effect of the sweets was observed in this study (Cummings et al., 2020). In a small-scale ecological momentary assessment study (*n* = 25), the consumption of sweets earlier in the day during abstinence even increased alcohol craving later in the day (Abrantes et al., 2022).

In a prospective study, AUD patients (*n* = 49) increased their consumption of chocolate and other sweets over 6 months of abstinence from alcohol, but this consumption pattern was not associated with reduced craving for alcohol (Stickel et al., 2016). In an older study, newly sober outpatients of an alcoholism treatment program (*n* = 64) were interviewed about dietary intake (calories, carbohydrates, sucrose, sugar added to beverages, protein, fats, vitamins and minerals) once weekly during the first month and then at wider intervals provided they remained sober. It appeared that those who stayed sober longer chose diets containing twice as much sugar added to beverages and more overall carbohydrates (*p* < 0.05) (Yung et al., 1983). Specifically for table sugar added to beverages, those who remained sober for more than 30 days used on average almost 3 times more sugar per cup of beverage than those who remained sober for less than 30 days (*p* < 0.01). It remains, however, to be clarified whether longer sobriety increased appetite for sugar or whether the choice of sugar influenced the patients’ ability to stay sober because initial data on the nutritional status prior to the study were not available (Yung et al., 1983).

In summary, upon acute discontinuation of addictive substances, supplementation with sugar could assist in mitigating symptoms of withdrawal. Most promising results were obtained in NRT-assisted smoking abstinence. With respect to AUD, two studies found preliminary support for the hypothesis that conceptualized sweets as an aid to abstinence (Stickel et al., 2016; Yung et al., 1983), whereas three other studies (Abrantes et al., 2022; Cummings et al., 2020; Krahn et al., 2006) using a sweet diet to reduce craving for alcohol showed weak or negative outcomes.

## Discussion

The present review has two main outcomes. Firstly, in smokers, glucose consumption prior to or during stop-smoking attempts seems to reduce signs of nicotine withdrawal relatively rapidly (McRobbie and Hajek, 2004; West et al., 1990, 1999) and to increase smoking abstinence induced by NRT (West and Willis, 1998; West et al., 2010). Secondly, SL AUD patients encounter more problems in attaining abstinence compared to SDL AUD patients (Garbutt et al., 2009). Interestingly, those with high cravings and the SL phenotype demonstrated higher rates of alcohol abstinent days compared to their SDL peers, and a positive response to naltrexone was associated with the SL phenotype and a high craving for alcohol (Garbutt et al., 2016). Additionally, SL was a positive effect modifier for a successful outcome of naltrexone treatment in AUD (Laaksonen et al., 2011). Together, these observations offer new opportunities to fine-tune and optimize pharmacotherapeutic approaches in addiction treatment.

The observed associations between sugar supplementation and SL on the one hand and dependence on nicotine and alcohol on the other are probably based on common underlying neurobiological mechanisms. Both sweet tastes and drugs of abuse activate mesolimbic dopamine and opioid pathways, known to be involved in motivation and reward (Volkow et al., 2008). Considering the overlap in reward circuits of sugar and nicotine, sugars may represent a functional substitute for nicotine by lowering the negative effect induced by smoking cessation. Sugar-induced mild activation of the dopaminergic pathway may mitigate withdrawal due to the diminished dopaminergic tone following smoking cessation. Indeed, the present results show that sugar supplementation diminished signs of smoking withdrawal (McRobbie and Hajek, 2004; West et al., 1990, 1999), facilitating successful outcomes in smoking cessation attempts.

Considering the efficacy of sugar supplementation in facilitating successful outcomes in smoking cessation attempts (McRobbie and Hajek, 2004; West et al., 1990, 1999), it is advocated to introduce sugar, and perhaps, as an add-on in smoking cessation therapy. Note that this advice is concordant with Alcohol Anonymous’ previous claim that individuals can reduce alcohol cravings by eating sweets (Alcoholics Anonymous, 2020). In addition, in treating AUD patients, clinicians have noted that many of the newly sober patients develop a carbohydrate appetite or ‘sweet tooth’. Some of them start the consumption of a large amount of cake, chocolate, ice cream and candies which they have not liked before. This effect is very common though not universal (Yung et al., 1983). Obviously, excessive consumption of high-caloric sweet commodities is not recommended as it may lead to health-threatening obesity and diabetes. Until more data are available, the addition of sweets should be restricted to the detoxification and early abstinence phase.

Another outcome of the current review is that SL/SDL phenotyping may be applicable in the treatment of AUD. The previously mentioned apparent overlap in reward pathways endorses the hypothesis that SL phenotype individuals are more sensitive to drug reward than SDL phenotype individuals. The latter may also be explained by the known association between sensitivity to drug rewards and impulsive behaviour. For instance, individuals who abused (and presumably liked) opiates, cocaine, nicotine and alcohol discounted delayed rewards to a greater extent than non-drug abusers (Bickel et al., 2014; MacKillop et al., 2011). With respect to ‘sugar reward’, it was demonstrated that healthy young adults with the SL phenotype showed greater impulsive choice (delay discounting task; *p* = 0.03), though it was not related to impulsive action (the go/no-go task; *p* = 0.79) (Weafer et al., 2014). Indeed, the results of genetic and descriptive studies confirm that SL subjects are genetically predisposed to substance abuse, such as AUD (Choi et al., 2017; Kampov-Polevoy et al., 2014, 2022; Lange et al., 2010) and CUD (Janowsky et al., 2003). More importantly, SL and SDL individuals respond differently in trials directed at abstinence from drinking. For instance, SL individuals showed higher responsiveness to naltrexone treatment of alcohol-dependent patients than SDL individuals, and, therefore, SL/SDL phenotyping could be used to stratify clinical samples in RCTs that evaluate new therapeutic approaches. Additionally, SL/SDL phenotyping may be used to predict the success of reduced drinking attempts and to estimate the level of additional support in those attempts (Garbutt et al., 2014). Finally, it is remarkable that the SD/SDL phenotype approach has not been tried in quit-smoking attempts or at least to stratify smokers according to this phenotype in RCTs. As a final note, we like to mention the cumbersome and labour-intensive assessment of the SD/SDL phenotypes. We therefore suggest developing simpler—clinically feasible—alternatives using, for example, widely available branded sweets or beverages.

## Conclusion

The findings outlined in this review provide promising clues to be implemented in addiction treatment protocols and offer one possible new explanation of the clinical observation that, for example, naltrexone is not effective for every patient. However, it is advocated to further investigate the putative link between sweet liking and alcohol dependence liability. This also applies to the use of sugar in addiction treatment as an add-on, such as a specific nutritional intervention using sweet commodities, to support detoxification and to prevent (early) relapse to a variety of addictive substances. In addition, SL/SDL phenotyping, along with other phenotypes, like novelty seeking and initial low level of response to alcohol (Schuckit and Smith, 2013) should be introduced to optimize the efficacy of treatment in AUD. Based on the perspectives currently provided, targeted interventions for high-risk individuals that prevent and support the treatment of AUD may be developed and improved.

### Study limitations

The current review has some important limitations, including a lack of replications of findings by different research groups, and a lack of adequate information on important subpopulations (ethnic groups, females, outpatients, polydrug users) and contextual confounders. For example, the innovative attempt to substitute the rewarding effects of addictive substances with sugars was mainly observed by one research group and in nicotine-dependent individuals only (i.e., West et al. 1990, 1998, 1999, 2010), and the promising results were only partly confirmed by other researchers (Helmers and Young, 1998; McRobbie and Hajek, 2004) but refuted by another research group (Harakas and Foulds, 2002). Moreover, the designs of the studies on sugar supplementation were rather weak with very short follow-up periods, with data not always adjusted for covariates, and there have been no new studies since 2010. With respect to SL phenotyping and the prediction of abstinence, the results are more consistent showing more craving, higher AUDIT-score and alcohol-related problems in SL phenotypes, although these results were not always confirmed (Bogucka-Bonikowska et al., 2001; Weafer et al., 2017; Wronski et al., 2007). Overall, the heterogeneity of the literature, in particular with respect to study designs and cohorts studied implies that the risk of bias in this review is relatively high.

In addition, the SL/SDL phenotyping approach was not evaluated in nicotine-dependent individuals. Furthermore, one may argue that the link between a familial history of an AUD (and other contextual determinants) and SL may be stronger than between a diagnosis of AUD and SL. Indeed, novelty seeking was shown in some studies to be a significant predictor of alcohol-related problems (Kampov-Polevoy et al., 2004, 2014, 2022; Lange et al., 2010). For instance, the odds of receiving an AUD diagnosis were shown to increase by some 11% for every one-point increase in the novelty-seeking score in SL but not in SDL (Kampov-Polevoy et al., 2004). However, the data in Tables 1 and 2 refer to well-defined AUD patients and clearly show an association between the two though not all studies presented have adjusted for confounding by introducing covariates, like alcohol-related problems in youth, gender, first-degree family history, craving for either alcohol or sweets and compulsivity.

Finally, we considered only two SL phenotypes (SL and SDL), whereas recent research has shown that there may be three distinct phenotypes with some participants having uncategorizable SL responses with (dis)liking astringency and bitterness (Armitage et al., 2023; Iatridi et al., 2019; Kavaliauskaite et al., 2023; Spinelli et al., 2021): (a) low sweet-likers/SDL, (b) extreme/high sweet-likers/SL and (c) medium/moderate/U-shaped SL who dislike very high and very low sugar solutions.

## Supplemental Material

sj-docx-1-jop-10.1177_02698811251319454 – Supplemental material for Sweet-liking and sugar supplementation as innovative components in substance use disorder treatment: A systematic review Supplemental material, sj-docx-1-jop-10.1177_02698811251319454 for Sweet-liking and sugar supplementation as innovative components in substance use disorder treatment: A systematic review by Jan van Amsterdam and Wim van den Brink in Journal of Psychopharmacology
